# A Global View of Cancer-Specific Transcript Variants by Subtractive Transcriptome-Wide Analysis

**DOI:** 10.1371/journal.pone.0004732

**Published:** 2009-03-06

**Authors:** Chunjiang He, Fang Zhou, Zhixiang Zuo, Hanhua Cheng, Rongjia Zhou

**Affiliations:** Department of Genetics and Center for Developmental Biology, College of Life Sciences, Wuhan University, Wuhan, People's Republic of China; Cleveland Clinic, United States of America

## Abstract

**Background:**

Alternative pre-mRNA splicing (AS) plays a central role in generating complex proteomes and influences development and disease. However, the regulation and etiology of AS in human tumorigenesis is not well understood.

**Methodology/Principal Findings:**

A Basic Local Alignment Search Tool database was constructed for the expressed sequence tags (ESTs) from all available databases of human cancer and normal tissues. An insertion or deletion in the alignment of EST/EST was used to identify alternatively spliced transcripts. Alignment of the ESTs with the genomic sequence was further used to confirm AS. Alternatively spliced transcripts in each tissue were then subtractively cross-screened to obtain tissue-specific variants. We systematically identified and characterized cancer/tissue-specific and alternatively spliced variants in the human genome based on a global view. We identified 15,093 cancer-specific variants of 9,989 genes from 27 types of human cancers and 14,376 normal tissue-specific variants of 7,240 genes from 35 normal tissues, which cover the main types of human tumors and normal tissues. Approximately 70% of these transcripts are novel. These data were integrated into a database HCSAS (http://202.114.72.39/database/human.html, pass:68756253). Moreover, we observed that the cancer-specific AS of both oncogenes and tumor suppressor genes are associated with specific cancer types. Cancer shows a preference in the selection of alternative splice-sites and utilization of alternative splicing types.

**Conclusions/Significance:**

These features of human cancer, together with the discovery of huge numbers of novel splice forms for cancer-associated genes, suggest an important and global role of cancer-specific AS during human tumorigenesis. We advise the use of cancer-specific alternative splicing as a potential source of new diagnostic, prognostic, predictive, and therapeutic tools for human cancer. The global view of cancer-specific AS is not only useful for exploring the complexity of the cancer transcriptome but also widens the eyeshot of clinical research.

## Introduction

It remains unknown how both intron removal and exon rearrangement are precisely regulated to produce correct proteomes in a cell type- or developmental stage-specific manner. Alternative splicing, the process by which the exons of primary transcripts can be spliced into different arrangements to produce structurally and functionally distinct mRNA and protein variants, is the most widely used mechanism to enhance the protein diversity of higher eukaryotic organisms. It has been estimated that 35%–94% of all human genes appear to undergo alternative splicing [1]–[7], suggesting that this mechanism has a major role in generating protein diversity. As sequence data continue to be generated from projects at an ever-increasing rate, the need for mining the data and constructing a repository for transcriptome information continues to grow as well.

In many pathological conditions, aberrantly spliced pre-mRNAs are generated because they escape the quality control mechanisms within cells (e.g. the nonsense mediated mRNA decay pathway) and are, therefore, translated into aberrant proteins involved in human diseases, including cancer [8]–[11]. It is estimated that approximately 60% of disease mutations in the human genome are splicing mutations [12], [13]. Currently, the analysis of cancer-specific alternative splicing is a promising step forward and potential source of new clinical diagnostic, prognostic, and therapeutic strategies. Evidence is accumulating that supports a connection between tumorigenesis and alternative splicing [14]–[18]. Using bioinformatic approaches, Xu and Lee discovered cancer-specific splice variants in 316 genes [19]. We previously identified testis-/testis cancer-specific splice variants using bioinformatic and experimental approaches [20].

Despite the growing interest in the impact of alternative splicing in various aspects of the biological processes, our understanding of alternative splicing is still scattered, and its general regulatory mechanisms, especially in tumorigenesis, are not well known [21], [22]. However, it is believed that cancer-specific splice variants could be involved in the etiopathogeny of many diseases and some might serve as diagnostic or prognostic markers. Moreover, the direct targeting of protein is probably an advantageous way of correcting cancer-associated splicing alterations. For example, the cancer-restricted splice variant protein could be used as the target for specific antibodies conjugated to tumor cell toxins for cancer treatments. The etiopathogeny concerning the cancer-specific AS and all related applications need to be explored further.

In order to advance our understanding of the biological significance of alternative splicing in human cancers, it is essential to systematically identify cancer-specific splicing events at the transcriptome level. In the present study, we performed a genome-wide analysis of alternative splicing in human cancer and normal tissues using an intersection/subtractive model consisting of the following steps: 1) identifying insertions or deletions in the alignments of expressed sequence tags (ESTs) to identify alternative splicing transcripts based on a previously described method [2], 2) the alignment of EST/genome to confirm the transcripts, and 3) obtaining the tissue-specific and alternatively spliced variants by subtractively cross-screening the alternatively spliced transcripts in each tissue. Our results distinguish distinctive patterns of cancer-specific alternative splicing and identify a large number of cancer- and tissue-specific splicing isoforms, which provides a global view of human cancer-specific alternative splicing in a large-scale approach and a potential source of new clinical diagnostic, prognostic, and therapeutic strategies for human cancer.

## Materials and Methods

### Data sources and filtration

Human EST data for both cancerous and normal tissues were drawn from the Cancer Genome Anatomy Project (CGAP) (http://cgap.nci.nih.gov/Tissues/LibraryFinder). The CGAP collects EST libraries from all over the world and provides good tissue information. All available EST libraries for both human cancer and normal tissues were downloaded from the CGAP libraries, Mammalian Gene Collection libraries, and Open Reading Frame EST Sequencing libraries. We sought to avoid mixing multiple tissues. Among these libraries, those signed ‘pooled’ were excluded because these procedures affect tissue classification. For normal tissue, ESTs were classified in accordance with the developmental stage information, and libraries without this information were not used. All EST and library data on different tissues that were used are listed in Tables 1 and 2.

**Table 1:** Numbers of libraries and ESTs in cancers.

Cancer types (27 types)	Libraries	ESTs
adrenal_cancer	3	10431
bone_marrow_leukemia	21	43943
brain_glioma	43	75546
brain_meningioma	7	860
brain_cancer	213	39845
breast_cancer	792	143423
cervical_cancer	32	48606
chondrosarcoma	15	49638
colorectal_cancer	803	185632
esophageal_cancer	17	15039
germ_cell_cancer	30	161682
head_and_neck_cancer	291	93978
kidney_cancer	87	74290
liver_cancer	62	117276
lung_cancer	220	126775
lymphoma	6	12458
muscle_tissue_cancer	26	83411
ovarian_cancer	162	98087
pancreas_insulinoma	1	33046
pancreatic_cancer	22	82994
primitive_neuroectodermal_cancer_of_CNS	23	72677
prostate_cancer	169	168151
prostatic_intraepithelial_neoplasia	5	9569
retinoblastoma	3	51568
skin_cancer	36	137803
stomach_cancer	244	94950
uterus_cancer	110	46624
**Total**	**3,443**	**2,078,302**

**Table 2:** Numbers of libraries and ESTs in normal tissues.

Tissue type (35 tissues)	Libraries	ESTs
bone-adult	2	1965
brain-adult	368	130619
brain-fetus	73	174889
brain-infant	11	73726
colon-adult	133	25757
colon-fetus	1	5
eye-adult	33	100173
eye-fetus	7	19659
heart-adult	10	3979
heart-fetus	13	62681
kidney-fetus	16	13947
liver-adult	11	29818
liver-fetus	22	142750
lung-adult	92	44457
lung-fetus	18	35607
mammary-gland-adult	331	68603
muscle-adult	10	70211
muscle-fetus	4	2235
ovary-adult	6	8126
pancreas-adult	10	24759
pancreas-fetus	2	5545
peripheral-nerve-adult	1	6571
peripheral-nerve-juvenile	1	9482
pituitary-gland-adult	6	8520
placenta-adult	358	268277
prostate-adult	127	53220
skin-infant	3	10319
spleen-adult	3	1841
spleen-fetus	1	1332
stomach-adult	67	10417
stomach-fetus	2	9
testis-adult	157	30549
thyroid-adult	78	13314
uterus-adult	10	35026
vascular-adult	5	8451
**Total**	**1,992**	**1,496,839**

All collection data were then dealt with in three procedures: repeat sequence masking to remove simple repeats in the dataset (program, repeatmasker; repeat database, repbase; girnst server:www.girinst.org), vector and contamination masking to clean the vector sequences (program, crossmatch; vector database, UniVec_Core; National Center for Biotechnology Information ftp server: ftp://ftp.ncbi.nih.gov/), and a final cleaning of short and rubbish sequences (program, seqclean from egassembler server: http://egassembler.hgc.jp). Any Alu repeats were included in, and the filtered ESTs were available for the following analysis.

### Computational procedures to identify cancer/tissue-specific alternative splicing

A basic local alignment search tool (BLAST) database was constructed for the ESTs of each tissue. Alternative splicing was analyzed based on a previous method [2]. Transcripts specific to tissue T were identified based on an intersection/subtractive model:
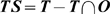



Where ***TS*** is the alternatively spliced transcripts specific to tissue T, ***T*** is all transcripts in tissue T, and ***O*** is all transcripts in the other tissues (**∩**, intersection).

Briefly, the three steps were as follows:

1. Tissue T's EST dataset was BLASTed against itself. The e-value was set to 1e-30. Gaps (insertion or deletion) in the ESTs were identified after alignment. Parameters to identify alternative splicing: the gap length, 10 bp; nucleotide identity, 95%.
2. Tissue T's ESTs were BLASTed against the ESTs of the other tissues. Parameters were the same as step 1.
3. Subtractive ESTs were identified as tissue T-specific ESTs by insertion/deletion comparisons after BLAST. Computer programs were written using the Perl language.

### EST/genomic sequence alignments, chromosome mapping, and splice site analysis

To decrease errors in EST alignments and determine the chromosomal loci of each gene, we localized ESTs to genomic sequences using BLAST-like alignment tools (http://genome.ucsc.edu). We used the default parameters and selected the best score results. The exon position on the chromosome was recorded for each transcript and used to determine splice sites and gene structure. Splice sites for both 5′ and 3′ exon/intron boundaries were aligned online via http://weblogo.berkeley.edu/logo.cgi. We allowed an error of 10 bp in the exon/intron boundary. Based on comparisons of EST/genomic alignments, two possible errors can be checked: (i) if the candidate EST in the same gene was not on the same chromosome and (ii) is the candidate EST in the same gene was not in the same locus on the chromosome. The reasons for these errors mainly included EST sequencing errors, pseudogenes, and multiple copy genes. The two cases were excluded as false positives in the final database.

### Function classification of alternative splicing

Each alternatively spliced EST was BLASTed to the RefSeq mRNA database (expectations 1e-30) to identify the corresponding genes. Using PANTHER (http://www.pantherdb.org/tools/genexAnalysis.jsp), these genes were clustered by the gene ontology (GO) process. We also searched the Entrez Gene Database to correct our results.

### Alternative splicing database construction

We input all prediction results into the local alternative splicing database. This database was constructed with MySql and programmed by Perl and CGI. All information such as gene ID, gene structure, EST accession, mRNA accession, gene information, and exon location on the chromosome were collected in the database.

## Results

### HCSAS: A database for cancer-specific alternative splicing

For analyzing cancer-specific alternative splicing, we carefully classified all available EST libraries into 35 distinct normal tissue classes and 27 types of cancer to avoid mixing multiple tissues. Our final classification consisted of 1,992 libraries with 1,496,839 ESTs for normal human samples and 3,443 libraries with 2,078,302 ESTs for cancer samples (Tables 1 and 2). Through computationally subtractive analysis, we detected 15,093 cancer-specific transcripts in 9,989 genes from the 27 types of cancer, and 14,376 normal tissue-specific transcripts in 7,240 genes from the 35 tissues (Tables 3 and 4), which cover the main types of human tumors and tissues. Cancer-specific transcript numbers per gene detected were 1 to 1.69 with an average of 1.51, whereas there were 1 to 6 normal tissue-specific transcripts with an average of 1.99 (Tables 3 and 4), indicating fewer alternative splicing events (cancer-specific) in cancer compared to normal tissues.

**Table 3:** Numbers of cancer-specific AS transcripts and their genes.

Cancer types (27 types)	Genes	Transcripts	Transcripts/Gene
adrenal_cancer	61	80	1.31
bone_marrow_leukemia	237	356	1.50
brain_glioma	485	720	1.48
brain_meningioma	1	1	1.00
brain_cancer	22	30	1.36
breast_cancer	550	757	1.38
cervical_cancer	316	428	1.35
chondrosarcoma	239	352	1.47
colorectal_cancer	397	578	1.46
esophageal_cancer	175	226	1.29
germ_cell_cancer	1307	2167	1.66
head_and_neck_cancer	135	182	1.35
kidney_cancer	467	716	1.53
liver_cancer	950	1410	1.48
lung_cancer	605	880	1.45
lymphoma	16	21	1.31
muscle_tissue_cancer	692	1044	1.51
ovarian_cancer	351	512	1.46
pancreas_insulinoma	45	76	1.69
pancreatic_cancer	426	643	1.51
primitive_neuroectodermal_cancer_of_CNS	659	1018	1.54
prostate_cancer	92	111	1.21
prostatic_intraepithelial_neoplasia	11	18	1.64
retinoblastoma	434	705	1.62
skin_cancer	939	1557	1.66
stomach_cancer	249	326	1.31
uterus_cancer	128	179	1.40
**Total**	**9,989**	**15,093**	**1.51**

**Table 4:** Numbers of normal tissue-specific AS transcripts and their genes.

Tissue types (35 tissues)	Genes	Transcripts	Transcripts/Gene
bone-adult	1	2	2
brain-adult	924	1613	1.75
brain-fetus	2231	5545	2.49
brain-infant	53	72	1.36
colon-adult	17	22	1.29
colon-fetus	1	2	2.00
eye-adult	582	907	1.56
eye-fetus	58	76	1.31
heart-adult	6	9	1.50
heart-fetus	100	146	1.46
kidney-fetus	110	142	1.29
liver-adult	335	961	2.87
liver-fetus	292	506	1.73
lung-adult	78	160	2.05
lung-fetus	110	152	1.38
mammary-adult	41	57	1.39
muscle-adult	250	384	1.54
muscle-fetus	18	27	1.50
ovary-adult	19	26	1.37
pancreas-adult	28	39	1.39
pancreas-fetus	23	33	1.43
peripheral-nerve-adult	18	23	1.28
peripheral-nerve-juvenile	18	23	1.28
pituitary-gland-adult	28	84	3.00
placenta-adult	1567	2902	1.85
prostate-adult	102	162	1.59
skin-infant	99	121	1.22
spleen-adult	2	2	1.00
spleen-fetus	1	1	1.00
stomach-adult	3	5	1.67
stomach-fetus	1	6	6.00
testis-adult	47	73	1.55
thyroid-adult	16	20	1.25
uterus-adult	51	63	1.2
vascular-adult	10	10	1.00
**Total**	**7240**	**14,376**	**1.99**

**Figure 1:**
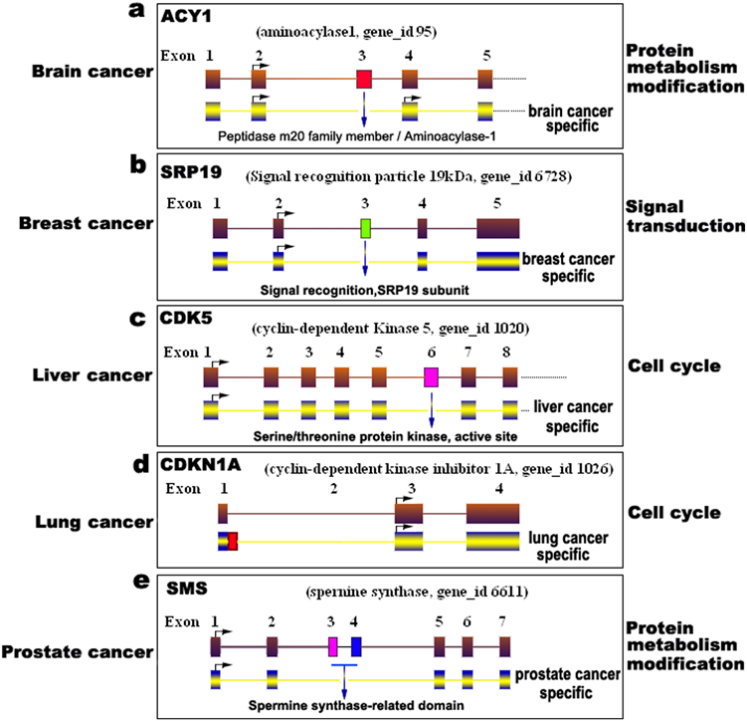
A schematic representation of cancer-specific alternative gene splicing. (a) Brain cancer (gene ACYl), (b) breast cancer (SRP19), (c) liver cancer (CDK5), (d) lung cancer (CDKN1A), and (e) prostate cancer (SMS). Cancer-specific isoforms are showed on the bottom in each panel. The biological processes of these transcripts (GO process) are indicated on the right. Deleted domains are shown with blue arrows. Arrows with a right angle indicate the start codon, ATG.

To facilitate future studies and referencing of alternatively spliced genes, for both human cancer and normal tissues, we constructed a human cancer- and normal tissue-specific alternative splicing database (HCSAS) based on our analysis, which was divided into two parts: cancer-specific (15,093 transcripts) and normal tissue-specific (14,376) alternative splicing. Of these cancer- or tissue-specific AS, approximately 70% are novel isoforms. For example, in brain cancer, because of the alternative splicing and deletion of domain of the peptidase m20 family member, the aminoacylase-1 gene (ACY1) was spliced to produce a brain cancer-specific transcript (Figure 1a), and alternative splicing occurs in the SRP19 gene to produce a breast cancer-specific transcript by an alternative deletion of exon 3 (Figure 1b). Similarly, in liver cancer, lung cancer, and prostate cancer, cancer-specific isoforms were detected in our subtractive screening (Figure 1c–e).

**Figure 2:**
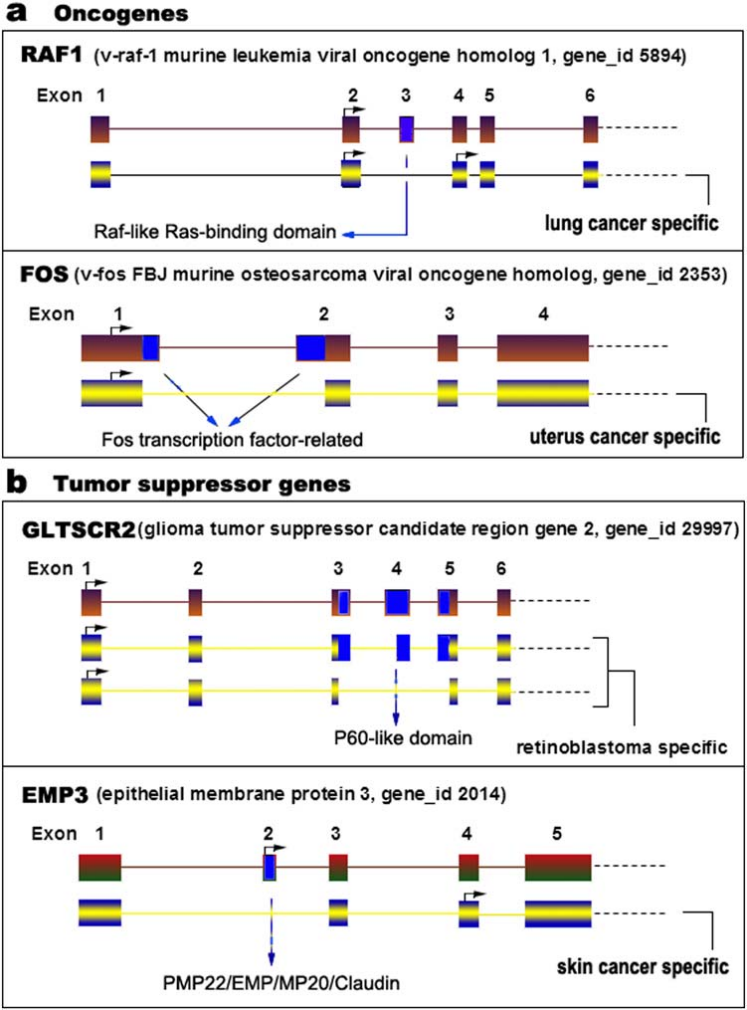
A schematic representation of cancer-specific alternative gene splicing. (a) Oncogene, (b) tumor suppressor gene. The alternative splicing of RAF1 generates a lung cancer-specific transcript, whereas the alternative splicing of FOS produces an uterus cancer-specific transcript. Tumor suppressor GLTSCR2 is alternatively spliced to produce two retinoblastoma-specific transcripts and EMP3 to generate a skin cancer-specific transcript. Deleted domains are shown with blue arrows. Arrows with a right angle indicate the start codon, ATG.

Furthermore, we systematically identified cancer-specific transcripts in both oncogenes and tumor suppressors. Thirty-nine oncogene isoforms and 38 tumor suppressor gene isoforms with cancer-specific AS events were detected (Table 5). For example, we identified a lung cancer-specific transcript in the oncogene RAF1 with a deletion of the Raf-like Ras-binding domain, an uterus cancer-specific transcript in oncogene FOS (Figure 2a), and a retinoblastoma-specific transcript in the tumor suppressor GLTSCR2, and a skin-cancer-specific transcript in the tumor suppressor EMP3 (Figure 2b).

**Table 5:** Oncogenes and tumor suppressors with cancer-specific AS events.

Gene ID	Symbol	Gene Description	AS
**Oncogenes**			
25	ABL1	v-abl Abelson murine leukemia viral oncogene homolog 1	1
3726	JUNB	jun B proto-oncogene	2
7409	VAV1	vav 1 oncogene	1
6757	SSX2	synovial sarcoma, X breakpoint 2	3
2130	EWSR1	Ewing sarcoma breakpoint region 1	3
2241	FER	fer (fps/fes related) tyrosine kinase (phosphoprotein NCP94)	2
369	ARAF	v-raf murine sarcoma 3611 viral oncogene homolog	1
4613	MYCN	v-myc myelocytomatosis viral related oncogene	2
2534	FYN	FYN oncogene related to SRC, FGR, YES	2
727735	unassigned	similar to TBC1 domain family member 3	1
51513	ETV7	ets variant gene 7 (TEL2 oncogene)	2
5894	RAF1	v-raf-1 murine leukemia viral oncogene homolog 1	2
4193	MDM2	Mdm2, transformed 3T3 cell double minute 2, p53 binding protein	1
4609	MYC	v-myc myelocytomatosis viral oncogene homolog	1
2353	FOS	v-fos FBJ murine osteosarcoma viral oncogene homolog	3
7410	VAV2	vav 2 oncogene	1
4194	MDM4	Mdm4, transformed 3T3 cell double minute 4, p53 binding protein	1
2118	ETV4	ets variant gene 4 (E1A enhancer binding protein, E1AF)	3
598	BCL2L1	BCL2-like 1	3
55885	LMO3	LIM domain only 3 (rhombotin-like 2)	1
3265	HRAS	v-Ha-ras Harvey rat sarcoma viral oncogene homolog	2
4893	NRAS	neuroblastoma RAS viral (v-ras) oncogene homolog	1
		**Total**	**39**
**Tumor suppressors**			
5934	RBL2	retinoblastoma-like 2 (p130)	1
3482	IGF2R	insulin-like growth factor 2 receptor	1
5925	RB1	retinoblastoma 1 (including osteosarcoma)	1
54984	unassigned	PIN2-interacting protein 1	1
4017	LOXL2	lysyl oxidase-like 2	3
29997	GLTSCR2	glioma tumor suppressor candidate region gene 2	3
2014	EMP3	epithelial membrane protein 3	1
672	BRCA1	breast cancer 1, early onset	2
54879	ST7L	suppression of cancerigenicity 7 like	2
51147	ING4	inhibitor of growth family, member 4	1
7982	ST7	suppression of cancerigenicity 7	1
51566	ARMCX3	armadillo repeat containing, X-linked 3	4
84695	LOXL3	lysyl oxidase-like 3	2
79961	DENND2D	DENN/MADD domain containing 2D	2
7157	TP53	cancer protein p53 (Li-Fraumeni syndrome)	1
7248	TSC1	tuberous sclerosis 1	1
54768	HYDIN	hydrocephalus inducing homolog	3
581	BAX	BCL2-associated X protein	1
1026	CDKN1A	cyclin-dependent kinase inhibitor 1A (p21, Cip1)	5
1029	CDKN2A	cyclin-dependent kinase inhibitor 2A	1
10263	CDK2AP2	CDK2-associated protein 2	1
		**Total**	**38**

**Figure 3:**
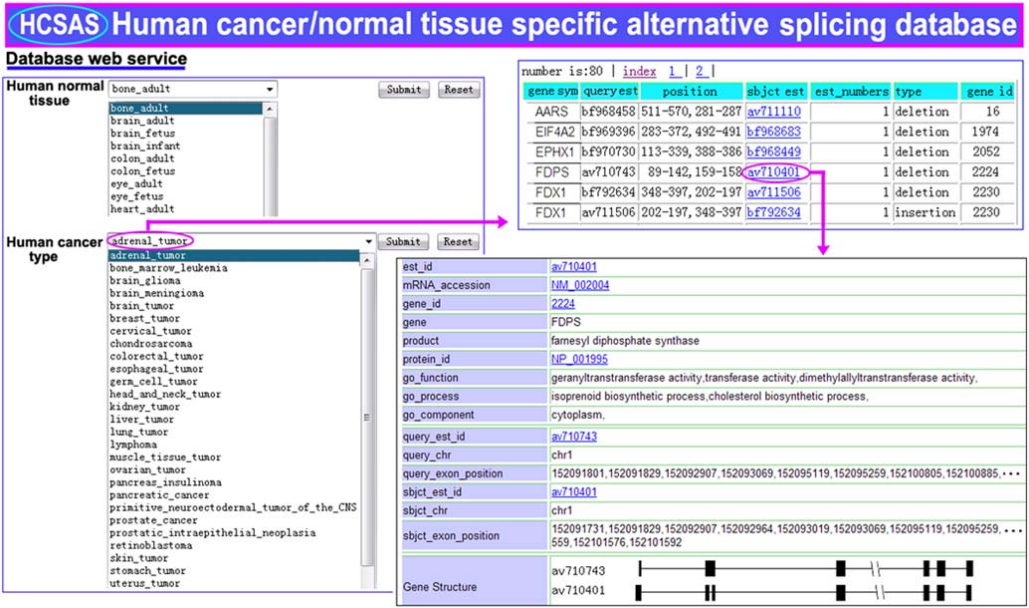
A database of cancer- and normal tissue-specific alternative splicing. An example page set from the database shows the details of an adrenal cancer gene, FDPS. The information includes the specific alternative splicing of both cancer and normal tissues, gene ID, gene structure, splicing sites, chromosome localization, DNA and protein sequences linked with the NCBI website, and GO process, function, and subcellular localization.

The HCSAS database presents a global overview of cancer-specific alternative splicing in humans and is essential for understanding tumorigenesis at a systematic level. The main information in this database includes the specific alternative splicing in both cancer and normal tissues, gene ID, gene structure, splicing sites, chromosome localization, DNA and protein sequences linked with the NCBI website, and GO process, function, and subcellular localization. An example page set shows the details of an adrenal cancer gene, FDPS (Figure 3). The HCSAS database can be accessed at http://202.114.72.39/database/human.html.

### Biased utilization of alternative splicing types in cancer

**Figure 4:**
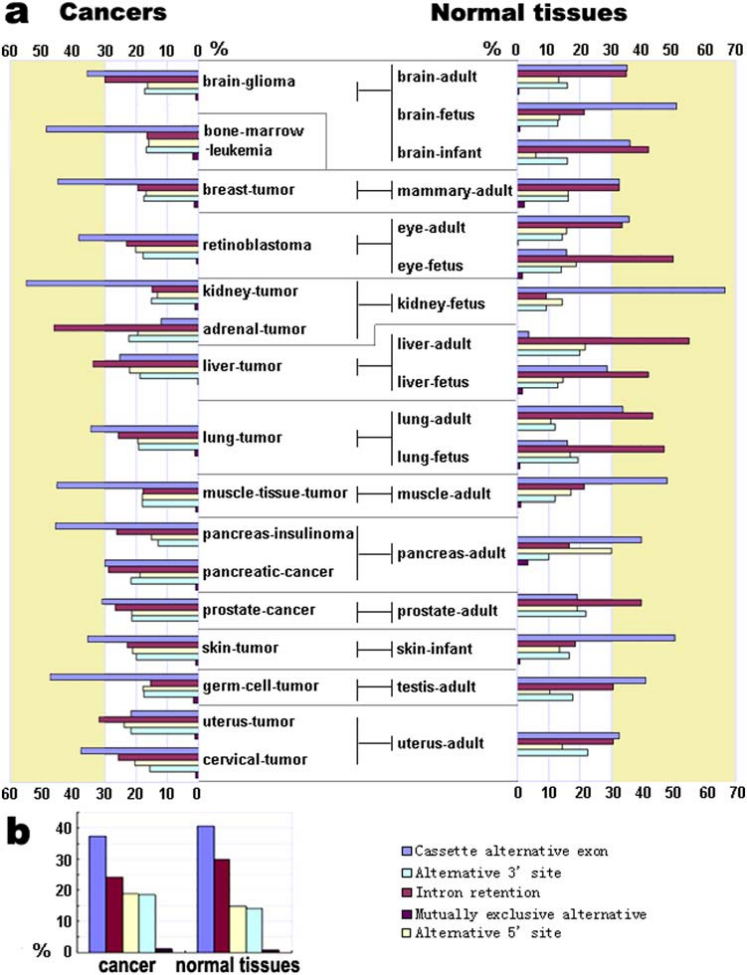
The frequencies (percentages) of the five types of cancer- and normal tissue-specific alternative splicing. (a) 16 types of human cancer and 17 normal tissues, (b) the average values between tumors and normal tissues. The five colors indicate the five types of tissue-specific alternative splicing: cassette alternative exon, alternative 5′ splice site, alternative 3′ splice site, intron retention, and mutually exclusive alternative exons. Yellowish regions indicate over 30% of the frequencies.

An examination of cancer-specific alternative splicing revealed a biased distribution of alternative splicing types in cancer. Both the alternative 3′ splice site and 5′ splice site were used more often in cancer; however, a lower proportion of intron retention and cassette alternative exon occurred in cancer tissues compared to normal tissues (Figure 4b). Moreover, alternative splicing types differ between different kinds of cancer (Figure 4a). For example, in liver cancer, breast cancer, and prostate cancer, intron retention decreased and cassette alternative exons increased significantly, whereas in uterus cancer and skin cancer, cassette alternative exons markedly decreased.

### Preference in the selection of alternative splice sites in cancer

**Figure 5:**
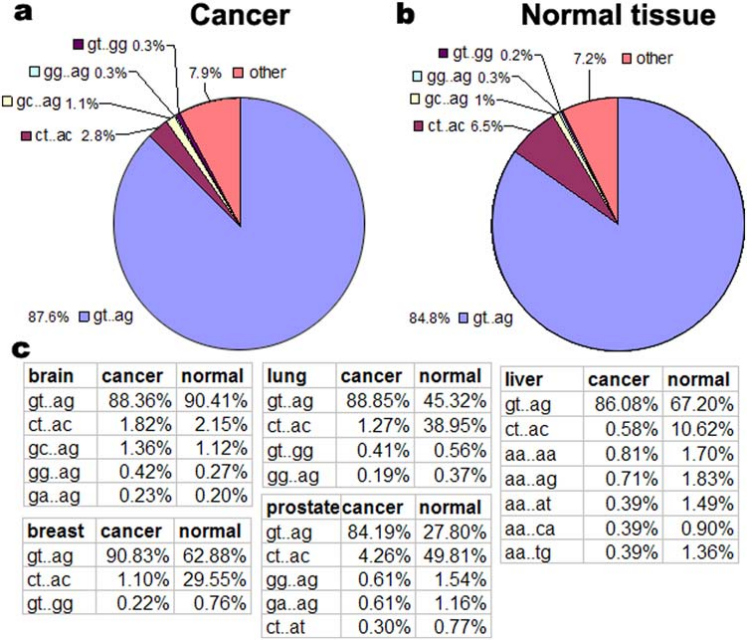
Percentages of the types of alternative splice sites. The splice sites include GT-AG, GC-AG, GG-AG, GT-GG, and the others (a) in human cancer (b) and normal tissues. (c) Percentage distribution of the splice sites in five types of cancer and normal tissues (brain, breast, lung, liver, and prostate).

To explore the preference/diversification of alternative splice sites in cancer, we analyzed all splice sites in the 27 types of cancer and 35 normal tissues by comparing each EST with its genomic sequence and mapping it onto the chromosome. We detected five basic donor-acceptor splice sites: GT-AG, CT-AC, GC-AG, GG-AG, and GT-GG, of which GT-AG are the most dominant sites. The others were classified into rare splice sites. We found that cancer uses rare splice sites and GT-AG more frequently, but less CT-AC compared to normal tissues (Figure 5a, b). Moreover, the selection of splice sites differs between different kinds of cancer (Figure 5c). For example, CT-AC sites are seldom used in breast cancer, liver cancer, lung cancer, and prostate cancer; in liver cancer, 5′ sites of rare splicing are almost AA.

### Association of cancer-specific alternative splicing of both oncogenes and tumor suppressor genes with cancer

**Figure 6:**
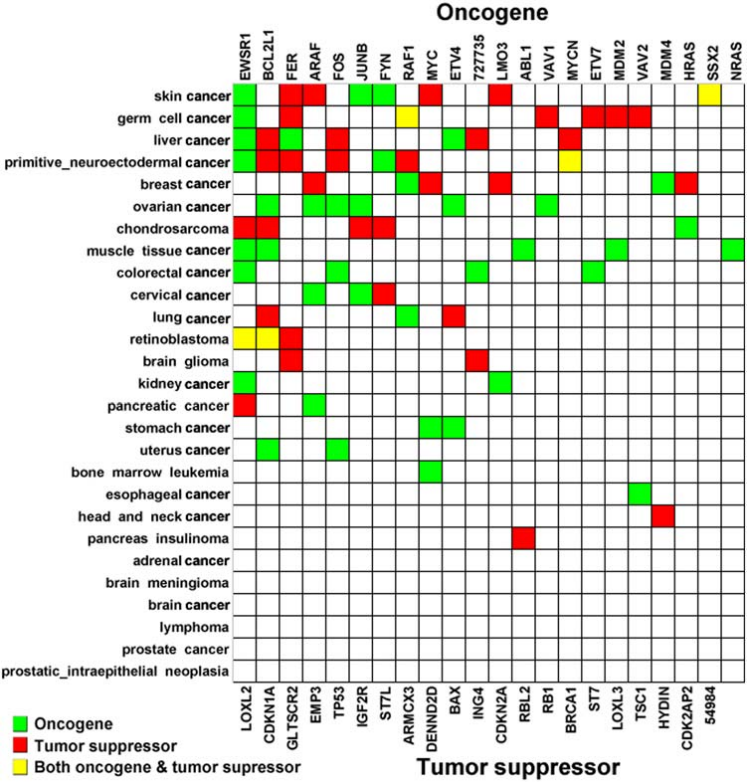
Distribution of oncogenes and tumor suppressors with cancer-specific alternative splicing in cancer. Blue squares indicate oncogenes, red squares indicate tumor suppressors, and yellow squares show both oncogenes and tumor suppressors.

Although both oncogenes and tumor suppressors are thought to be vital factors in tumorigenesis, we sought to identify cancer-specific variants and their possible involvement in cancer. We observed that oncogenes with cancer-specific AS are more often present in ovary cancer (6 oncogenes) and muscle cancer (5 oncogenes), whereas tumor suppressor genes with cancer-specific AS are more frequent in germ cell cancer (6), skin cancer (5), and primitive neuroectodermal cancer (5) (Figure 6). Some oncogenes and tumor suppressors with cancer-specific alternative splicing, such as EWSR1, CDKN1A, and GLTSCR2, are present in more types of cancer. Moreover, neither oncogenes nor tumor suppressors with cancer-specific AS were detected in brain cancer, prostate cancer, adrenal cancer, or lymphoma. This distribution bias for cancer-specific AS implies that the cancer-specific alternative splicing of both oncogenes and tumor suppressor genes is associated with specific cancer types.

### Biological relevance of the cancer-specific transcripts in the diversification of protein functions

**Figure 7:**
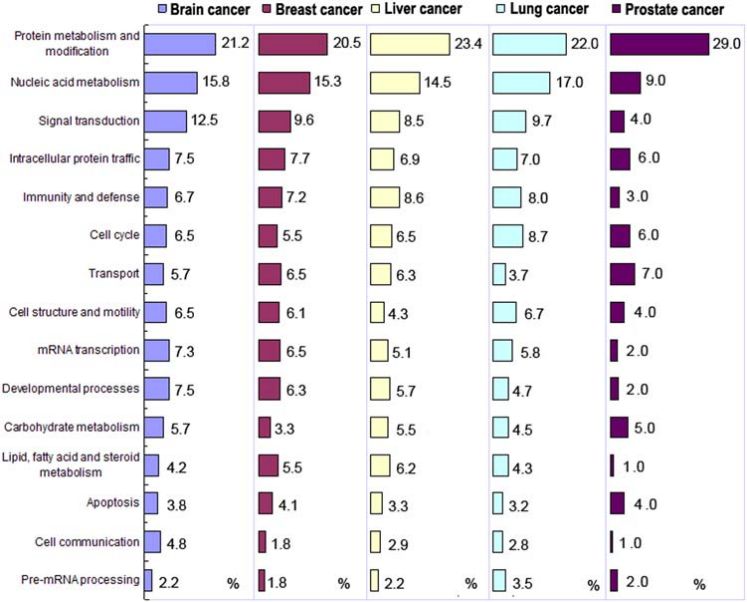
Biological processes of alternatively spliced transcripts specific to cancer. The five cancer types are brain, breast, liver, lung, and prostate cancer. The numbers indicate the percentages for each process in the cancer. The GO process classification is based on the PANTHER (http://www.pantherdb.org/tools/genexAnalysis.jsp).

The cancer-specific transcripts were classified based on gene function by searching the RefSeq database and GO. We classified 15,093 cancer-specific transcripts from 9,989 genes into 15 function groups. Protein metabolism and modification, and nucleic acid metabolism are the most prevalent functional processes in cancer. However, the function groups of these cancer-specific transcripts differ in different cancers. For example, the least common process in breast cancer is pre-mRNA processing, whereas the function groups of cell communication and lipid, fatty acid, and steroid metabolism are seldom found in prostate cancer (Figure 7).

## Discussion

The complexity of the transcriptome has been underestimated. In this paper, we described the transcriptome-wide identification and characterization of cancer-specific and alternatively spliced variants in human cancer based on a global view of cancer-specific alternative splicing developed by subtractive transcriptome-wide analysis. Based on an intersection/subtractive model, we have developed an analysis method for precisely screening cancer-specific alternative splicing. The EST sequences were aligned first, compared with their genomic sequences, and then mapped onto chromosomes. These procedures eliminated many EST errors, pseudogene, and multiple-copy/repeat gene problems when data were from diverse EST databases. Finally, the alternatively spliced transcripts were subject to the subtractive screening of a tissue versus all other tissues, and these analyses finally yielded cancer-specific transcripts. We identified a large number of cancer- / normal tissue-specific transcripts. Beyond all doubt, this is an abundant resource for research and the development of new diagnostic, prognostic, predictive, and therapeutic tools against human cancer. Furthermore, these resources are integrated into an available database. The HCSAS database presents a global overview of cancer-specific alternative splicing in humans and is essential for understanding tumorigenesis at a systematic level.

There are two main approaches for the global analysis of alternative splicing. First, based on the availability of sequenced genomes and large databases of sequenced transcripts (ESTs and cDNAs), alternative splicing events may be searched through reciprocal transcript alignments and alignments to genomic sequences. Several analyses in this manner have been reported [6], [23]–[29]. Because of its major limitation of EST coverage bias, a microarray-based technology has been developed to search for the alternative splicing events [3], [30]–[36]. Large sets of oligonucleotide probes may be designed specifically for individual exons and/or splice junction sequences, which allow the identification of new AS events. Here we have further developed a systematic method to search for cancer- or tissue-specific AS events in transcriptomes based on the intersection/subtractive screening analyses of transcriptomes, which is especially useful for identifying cancer/tissue-specific variants. Using this method, large numbers of cancer-specific isoforms were identified for the main human cancers. Nevertheless, these transcripts need to be further confirmed for their cancer/tissue specialization. RT-PCR technology and/or microarrays may be useful screening tools for this analysis.

Based on the transcriptome-wide analysis, we did observe special patterns of cancer-specific alternative splicing. 1) Less cancer-specific AS events occur in cancer compared to normal tissues. 2) Cancer possesses distribution bias for alternative splicing types. 3) Cancer uses rare splice sites and GT-AG more frequently, but less CT-AC compared to normal tissues. 4) The selection of splice sites differs between different kinds of cancer. 5) The cancer-specific alternative splicing of both oncogenes and tumor suppressor genes is associated with the specific cancer type. And finally, the functional groups of these cancer-specific transcripts differ in different cancers, indicating that individual cancers prefer combination controls of pathways in preference of using AS in tumorigenesis. These special features of human cancers indicate that 1) the cellular splicing machinery is changed during the transformation from normal to cancerous, 2) alternative splicing plays an important role during tumorigenesis, and 3) individual cancers have unique regulatory combinations at the alternative splicing level, which further support the prediction that approximately 60% of disease mutations in the human genome are splicing mutations [12], [13]. Our data includes the discovery of many novel splice forms of cancer-associated genes and alternative-splicing patterns in cancer, and it suggests a significant new direction for human cancer research. We strongly advise the use of cancer-specific alternative splicing as a potential source of new diagnostic, prognostic, predictive, and therapeutic tools against human cancer. The global view of cancer-specific AS is not only useful for exploring the complexity of the cancer transcriptome, but it also widens the eyeshot of clinical research.
